# Potential Effects of *Akkermansia Muciniphila* in Aging and Aging-Related Diseases: Current Evidence and Perspectives

**DOI:** 10.14336/AD.2023.0325

**Published:** 2023-12-01

**Authors:** Shi-Yu Zeng, Yi-Fu Liu, Jiang-Hua Liu, Zhao-Lin Zeng, Hui Xie, Jiang-Hua Liu

**Affiliations:** ^1^Department of Metabolism and Endocrinology, The First Affiliated Hospital, Hengyang Medical School, University of South China, Hengyang 421001, Hunan, China.; ^2^Department of Orthopedics, The First Affiliated Hospital, Hengyang Medical School, University of South China, Hengyang 421001, Hunan, China.; ^3^Department of Orthopedics, Xiangya Hospital, Central South University, Changsha, Hunan 410008, China.; ^4^Movement System Injury and Repair Research Center, Xiangya Hospital, Central South University, Changsha 410008, Hunan, China.; ^5^Department of Urology, The First Affiliated Hospital of Nanchang University, Nanchang 330006, Jiangxi, China.

**Keywords:** gut microbiome, *Akkermansia muciniphila*, intestinal barrier, short-chain fatty acids, extracellular vesicles, aging, age-related diseases

## Abstract

*Akkermansia muciniphila* (*A. muciniphila*) is an anaerobic bacterium that widely colonizes the mucus layer of the human and animal gut. The role of this symbiotic bacterium in host metabolism, inflammation, and cancer immunotherapy has been extensively investigated over the past 20 years. Recently, a growing number of studies have revealed a link between *A. muciniphila*, and aging and aging-related diseases (ARDs). Research in this area is gradually shifting from correlation analysis to exploration of causal relationships. Here, we systematically reviewed the association of *A. muciniphila* with aging and ARDs (including vascular degeneration, neurodegenerative diseases, osteoporosis, chronic kidney disease, and type 2 diabetes). Furthermore, we summarize the potential mechanisms of action of *A. muciniphila* and offer perspectives for future studies.

## Introduction

Longer life expectancy has long been a human dream; thanks to medical advances, improvements in the public health system, and economic development, this goal has been constantly surpassed. It is expected that by 2050, the number of people aged over 60 years will exceed 2 billion worldwide [1]. Age is undoubtedly the most life-threatening factor in older adults. Aging is characterized by cellular aging and degeneration of organ function, which increase susceptibility and decrease tolerance to aging-related diseases (ARDs) in the older adult population [2]. However, the pathological mechanisms of ARDs have not been adequately elucidated, which greatly limits the efficacy of treatment for these diseases in clinical practice.

The human microbiome is currently the most dynamic area of research, focusing on the gut, as it houses the majority of microorganisms. The gut microbiome is composed of over 10^14^ microorganisms, approximately 100 times more than human genes; hence, it is called the ‘second genome' [3, 4]. The gut microbiota usually varies among individuals because of differences in genetics, dietary habits, and environmental factors [5]. Notably, the composition of the gut microbiota changes gradually with age, making it significantly different between older and younger individuals [6]. Furthermore, these changes in the gut microbiota are believed to be closely associated with ARDs in the older adult population and may help address ARDs and increased body fragility[7].

*Akkermansia muciniphila* (*A. muciniphila*) is a member of *Verrucomicrobia* (phylum), which uses mucin as an energy source and is considered the "next generation of probiotics" [8]. Since its first characterization in 2004, this symbiotic bacterium has been associated with immune-related and metabolic disorders in humans [9]. As research progresses, exploration is shifting from correlation to causation [10]. Notably, recent studies have shown that the abundance of *A. muciniphila* in the feces of older mice is significantly lower than that in the feces of younger mice [11]. In addition, *A. muciniphila* is more prevalent in the gut microbiota of healthy older adults or long-lived populations (centenarians) compared to non-healthy older adult populations [12, 13], suggesting that upregulation of *A. muciniphila* abundance may be strongly associated with an increased healthy lifespan, making the impact of *A. muciniphila* on aging and ARDs a promising and far-reaching topic. Therefore, we present a review in this direction.

## Characteristics of *A. muciniphila*

*A. muciniphila* is an oval gram-negative bacterium, with only 1% of cells surviving after 48h of exposure to air [14]. In total, over 40 *A. muciniphila* subtypes have been identified, and these strains have been classified into four main phylogroups (AmI, AmII, AmIII, and AmIV) based on the maximum likelihood phylogenetic analysis of single nucleotide polymorphisms [15, 16]. Notably, the highest occurrence of AmI has been reported in humans, pigs, and mice. Within each phylogroup, the average nucleotide identity across genomes is 97.2-100%, compared to 86.8-91.5% between phylogroups [15]. Structurally, the AmII/AmIII genomes carry a greater number of CAZymes than that of AmI, particularly glycosyltransferase family 4, making their roles in carbohydrate and substrate metabolism more significant [15]. In terms of physiological function, the AmI strain has the shortest doubling time, the AmII strain has the best tolerance to oxygen, and the AmIV strain has the highest adhesion to intestinal epithelial cells [17]. Evidently, there are differences, albeit weakly, among the different isoforms of *A. muciniphila*. However, the extent to which these differences affect function remains unknown and requires close attention in subsequent studies.

ATCC BAA-835 is a representative strain of *A. muciniphila* belonging to AmI, whose complete genome consists of a 2,664,102 bp circular chromosome with 2,176 protein-coding sequences, of which 1,408 are thought to possess putative functions [18]. These proteins contain multiple glycosyl hydrolases, proteases, and sulfatases, which provide a structural basis for their mucin degradation capacity [18]. By fermenting mucin, free sulfate is released, and short-chain fatty acids (SCFAs) are produced in the intestine. Previous studies found that after 7 days, a single gavage of 10^9^ ATTC BAA-835 cells could colonize the intestines of germ-free mice, with the highest abundance in the cecum [19]. This finding implies that *A. muciniphila* has a good gastric acid tolerance and provides a basis for strain transplantation in clinical and preclinical studies. The mucus layer in the mammalian intestine separates the intestinal epithelium from the micro-organisms. Notably, *A. muciniphila* can penetrate this layer of mucus and directly colonize the surface of the intestinal epithelium [19].

In human feces, the relative abundance of *A. muciniphila* is approximately 1-3% of the total number of bacterial cells (1g of feces contains 10^8^-10^9^cells of cells) [20, 21]. The presence of *A. muciniphila* in breast milk likely allows this bacterium to colonize the intestines early in human life [22]. After one year of development, the abundance of this commensal bacterium can reach the levels observed in adults and gradually decrease with age [20]. In addition to being present in the human gut, *A. muciniphila* can colonize other species, including mice [23], horses [24], rabbits [25], guinea pigs [26] and pythons [27]. The prevalence of colonization in living organisms provides a basis for finding suitable animal models for studying human diseases and crosstalk in *A. muciniphila*.

A growing body of evidence supports the involvement of *A. muciniphila* in regulating host metabolism and immune homeostasis. However, the exploration of the mechanisms of action in the current research is extensive and lacking in depth, with the following main models: On one hand, the function of *A. muciniphila* is dependent on its SCFAs production capacity and intestinal barrier maintenance capacity, which effectively reduces systemic inflammation in the host [28, 29]. On the other hand, the role of *A. muciniphila's* outer membrane proteins (e.g., Amuc_1100), secreted proteins (e.g., P9), and secreted extracellular vesicles cannot be overlooked, as these components replicate a part of *A. muciniphila*'s function [29-31].

## *A. muciniphila* and aging

In general, the abundance of *A. muciniphila* in the human gut decreases with age [20]. However, several recent studies have found that *A. muciniphila* is significantly enriched in the gut of healthy and long-lived older adults [12, 13, 32]. Furthermore, another study supported the health benefits of *A. muciniphila* in centenarians. This study observed a significant reduction in *A. muciniphila* in the feces of centenarians as their health gradually deteriorated [33]. This suggests that *A. muciniphila* plays a key role in maintaining healthy aging.

Preliminary evidence from preclinical studies has revealed the anti-aging potential of *A. muciniphila*. Van et al. found that supplementation with *A. muciniphila* increased the thickness of the mucus layer in the gut of senescent mice and improved their systemic immune status [34]. Transplantation of *A. muciniphila* into prematurely aged mice extended their healthy lifespan, which may be related to the upregulation of secondary bile acid levels [13]. Furthermore, oral administration of *A. muciniphila* improved gut integrity and homeostasis in aged mice and restored cognitive function and muscle atrophy, while extending the healthy lifespan[11]. Similarly, a study exploring immune cell function and redox status in aged mice found that supplementation with *A. muciniphila* improved immune cell chemotaxis, phagocytosis, NK activity, and proliferative capacity and reduced oxidative stress parameters and pro-inflammatory cytokines [35]. More encouragingly, a study by Ma et al. found that glucose sensitivity, hepatosplenomegaly, inflammation, antioxidant capacity, and intestinal barrier function improved in aged mice after supplementation with *A. muciniphila* [28]. This implies that *A. muciniphila* may exert beneficial regulatory effects on host metabolism and immune function during aging.

The link between healthy diet and longevity has long been a topic of great interest. Recently, an increasing number of plant-based supplements have been shown to exert anti-aging effects [36]. Curcumin, ginseng, and apple polyphenols have been identified to increase the lifespan of fruit flies and Caenorhabditis elegans [37-42]. Beta-Carotene is thought to modulate telomerase activity in old age [43]. Of note, we found that almost all these anti-aging plant supplements increased the abundance of *A. muciniphila* to varying degrees [44-47]. However, it has not been possible to determine whether the anti-aging effects of these plant supplements are associated with an increased abundance of *A. muciniphila*. However, these studies collectively support the antiaging potential of *A. muciniphila* and introduce new ideas in aging-related research.

## *A. muciniphila* and age-related diseases

### A. muciniphila and vascular degeneration

Aging has an impact on the heart and arterial system, exemplified by pathological changes that can include hypertrophy, reduced left ventricular diastolic function and systolic reversal capacity, increased arterial stiffness, and impaired vascular endothelial function [48, 49]. This ultimately leads to an increased risk of cardiovascular diseases, such as atherosclerosis, hypertension, myocardial ischemia, and cerebrovascular embolism [48]. Therefore, although these disorders are more often considered to be caused by metabolic disorders, their association with aging should not be overlooked.

The crosstalk between *A. muciniphila* and vascular lesions has also been reported. In ApoE-deficient mice, *A. muciniphila* ameliorates metabolic endotoxemia-induced inflammation by reducing intestinal permeability, leading to the remission of atherosclerosis [50]. Similarly, flavonoids in sunflowers have been shown to be beneficial against atherosclerosis, and *A. muciniphila* abundance can be significantly upregulated in atherosclerotic mice[51]. Statins are routinely used to treat coronary artery disease (mainly atherosclerosis). A study exploring the relationship between gut microbiota and drug response in patients with coronary artery disease noted a significant reduction in the abundance of *A. muciniphila* and *Lactobacillus* in those who responded poorly to statins [52]. This implies that increasing the abundance of these probiotics may help patients with coronary artery disease to effectively control their blood lipids.

Abdominal aortic aneurysm (AAA) is an age-related degenerative lesion characterized by abnormal dilatation of the aorta due to degeneration of the arterial wall [53]. Microbial macrogenome sequencing revealed alterations in the gut microbiome in a mouse model of AAA in which *A. muciniphila* abundance was nearly depleted[54]. Subsequently, He et al. explored whether force-fed *A. muciniphila* exerted a protective effect in AAA mice. Consistent with our expectations, oral administration of *A. muciniphila* suppressed AAA formation by increasing the diversity of the gut microbiota and modulating the expression of peripheral immune factors [55]. Aging is also a high-risk factor for vascular calcification, with the latter leading to thickening of the arterial wall and reduced vascular compliance, which can induce a range of adverse cardiovascular events [56, 57]. A recent study exploring the role of propionate and gut microbiota in vascular calcification showed that transplantation of *A. muciniphila* alleviated vitamin D3- and nicotine-induced vascular calcification in mice [58]. Therefore, it is reasonable to believe that there is a crucial link between *A. muciniphila* and age-related vascular degenerative lesions.

### A. muciniphila and neurodegenerative diseases

Among diseases associated with aging, neuro-degeneration and the associated cognitive dysfunction are extremely important because they affect the quality of life to a large extent[59]. Aging is also associated with an increased risk of neurodegenerative diseases [60]. In the United States, the prevalence of Alzheimer's disease (AD) can reach 50% in people aged ≥95 years [61]. Globally, Parkinson's disease (PD) is second only to AD in prevalence, affecting more than 3% of the population aged ≥80 years [62]. Similarly, age > 60 years is considered a high-risk factor for amyotrophic lateral sclerosis (ALS) [63]. However, effective treatment strategies for age-related degeneration are often lacking, contributing to its irreversible progression. However, the rapid development of the field of the brain-gut axis has led to a greater understanding of neurodegenerative diseases.

Evidence has been presented regarding the association between *A. muciniphila* and AD. For example, Oral administration of *A. muciniphila* reduces Aβ plaque deposition in the brains of APP/PS1 mice, thereby alleviating cognitive impairment and anxiety [64]. *A. muciniphila* demonstrated similar alleviative effects in an AD-like rat model induced with AlCl3 and D-galactose, which was hypothesized to be associated with the modulation of gut microbiota composition and improvement in peripheral circulating metabolism [65]. Additionally, the potential modulatory effects of gut microbes on ALS were explored in a pivotal study. Serum and cerebrospinal fluid levels of nicotinamide (NAM) were upregulated in mice with ALS supplemented with *A. muciniphila*, which was associated with an increase in neurons in the spinal cord and improvement in motor symptoms in mice [66]. Overall, these results support the idea that *A. muciniphila* is beneficial in AD and ALS despite the lack of corroborating evidence from clinical practice.

Multiple 16S rRNA gene sequence assays in patients with PD have also revealed a link with the gut microbiome [67, 68]. These studies have consistently found that *A. muciniphila* is significantly enriched in patients with PD. Moreover, another study found that the prevention of dopaminergic neuronal death in the substantia nigra by Korean red ginseng was accompanied by a corresponding reduction in the number of *A. muciniphila* in the intestines of PD mice [69]. More importantly, recent studies have shown that over-colonization of *A. muciniphila* may accelerate the progression of PD as it promotes the aggregation of α-synuclein in intestinal endocrine cells [70]. This indicates that *A. muciniphila* has the potential to be used as a biomarker for assessing PD progression. Despite the lack of large-scale supporting data, evidence suggests that over-colonization by *A. muciniphila* is associated with thinning of the mucus layer and deterioration of the intestinal barrier [71-73]. This suggests that despite the large body of literature pointing towards *A. muciniphila* as beneficial to the health of the host, the possibility of its harmful function in certain specific environments cannot be ignored. However, it seems premature to conclude that *A. muciniphila* is associated with deterioration of PD. This is because there are still several concerns that remain unresolved. First, it is still an open question whether animal models can mimic human disease well; after all, *A. muciniphila* abundance is not significantly increased in most PD models [74, 75]. Second, whether multiple taxa, rather than individual strains, work together to influence PD progression is not clear. Third, it is possible that the increased colonization by *A. muciniphila* in the gut of patients with PD is compensatory and exerts a beneficial effect. Fourth, the sensitivity of *A. muciniphila* to drugs is also an important confounding factor that may contribute to the differences in the abundance of *A. muciniphila* in different ARDs. Finally, variations in the gut transit time may confound disease-associated microbiome characteristics, which may lead to interindividual differences in *A. muciniphila* abundance [76]. Therefore, extensive investigation is required to answer these questions.

### A. muciniphila and osteoporosis

Osteoporosis is a natural consequence of aging and inevitably places a huge burden on public health as the population ages. Importantly, there are still concerns regarding compliance with- and side effects of current treatment drugs [77]. Alterations in the gut microbiota have recently been explored in both osteoporotic populations and animal models. The results showed that the amount of *A. muciniphila* in the feces of osteoporosis groups was significantly reduced relative to that in healthy controls [78-80]. More critically, our study found that transplantation of child-derived fecal bacteria with live *A. muciniphila* or *A. muciniphila*-derived extracellular vesicles (AmEVs) into osteoporotic mice increased osteogenic activity and inhibited osteoclast formation [23]. In addition, *A. muciniphila* appears to have therapeutic effects against osteoporotic complications. We found that transplantation of *A. muciniphila* promotes H-type angiogenesis and fracture healing by alleviating systemic inflammation in mice [81]. Collectively, these results show that *A. muciniphila* can maintain bone mass and strength through multiple pathways, providing a new perspective for the management of osteoporosis.

### A. muciniphila and chronic kidney disease

Aging is accompanied by an increased incidence of chronic kidney disease (CKD), which in turn can lead to organ degeneration and increased physical fragility in older populations [82, 83]. This reveals the critical role of CKD in the older adult population and highlights the importance of relevant prevention and treatment strategies. Increased urea concentrations in CKD can lead to dysbiosis of the gut microbiota, which can exacerbate the production of intestinal toxins and increase intestinal permeability, changes that in turn exacerbate CKD [84]. Therefore, strategies that target intestinal microbiota are promising for mitigating the progression of CKD. In previous studies, the administration of an iron citrate complex in CKD rats improved creatinine clearance and hyperphosphatemia [85]. The simultaneous administration of the complex led to alterations in gut microbial composition, such as an increase in the number of *A. muciniphila*. Additionally, 16S rRNA gene sequencing in 50 patients with CKD and 22 healthy individuals showed that the CKD group possessed a lower abundance of *A. muciniphila*, which was negatively correlated with interleukin 10 levels [86]. Recent evidence supports the benefits of *A. muciniphila* in CKD. In that study, a 5/6 nephrectomy rat model showed significantly improved renal function and alleviated interstitial fibrosis following oral supplementation with *A. muciniphila* [87]. In conclusion, these preclinical studies have characterized the anti-CKD effects of *A. muciniphila*. However, clinical studies with larger sample sizes are required to confirm these findings.

### A. muciniphila and type 2 diabetes

Type 2 diabetes (T2D) is caused by impaired glucose metabolism due to increased insulin resistance in peripheral tissues. The risk of developing T2D increases with age, with the majority of patients aged > 65 years [88]. Senescence-related aseptic inflammation, impaired adipocyte progenitor cell function, and SASP factors secreted by senescent cells (e.g., activin A and TNF-α) are all associated with insulin resistance [89]. In addition, studies have shown that the phenotype of diabetes, including glucose tolerance and insulin sensitivity, can be improved with anti-aging drugs [90]. Therefore, T2D is often considered an ARD.

T2D and obesity are inextricably linked, with reduced A. *muciniphila* colonization in the gut of individuals with T2D and obesity. For the first time, insulin resistance in mice improved after increasing the abundance of *A. muciniphila* through probiotic supplementation [91]. This provides preliminary evidence of the crosstalk between T2D and *A. muciniphila*. Clinical evidence further validated this speculation as the amount of A. muciniphila in the feces of prediabetic patients was significantly lower than that in patients with normal glucose tolerance [92]. Subsequently, to demonstrate the antidiabetic potential of *A. muciniphila*, the investigators administered live *A. muciniphila* or purified membrane proteins directly by gavage to mice on a high-fat diet and observed an improved diabetic phenotype and reduced adipose tissue inflammation [29, 93]. Encouragingly, in a randomized controlled clinical trial involving 32 obese volunteers, *A. muciniphila* showed good tolerability and improved insulin sensitivity [94]. Notably, in this study, pasteurized *A. muciniphila* also improved insulin sensitivity and reduced insulinemia, total cholesterol, body weight, and fat mass [94]. This is a key finding, as pasteurized *A. muciniphila* avoids the safety concerns associated with the use of live *A. muciniphila*. This body of evidence holds promise for the application of *A. muciniphila* to obesity-related T2D in the foreseeable future.

Although 70-80% of people with T2D are obese, a small proportion of the population is underweight or has normal weight[95, 96]. Therefore, it is worth exploring whether *A. muciniphila* exerts similar antidiabetic effects in T2D lean individuals. The number of *A. muciniphila* in the feces of lean individuals with T2D was found to be significantly lower than that in lean individuals without T2D [97]. Furthermore, administration of *A. muciniphila* protected mice from high sucrose-induced adverse glucose tolerance impairment by reducing serum 3β-chenodeoxycholic acid and increasing insulin secretion [97]. In contrast, colonization of the gut by *A. muciniphila* was similarly reduced in patients with refractory T2D and negatively correlated with hemoglobin A1c [98]. However, direct evidence that *A. muciniphila* supplementation alleviates the diabetic phenotype in these specific T2D populations is lacking.

Overall, apart from the controversial evidence on PD, an increased abundance of *A. muciniphila* is often associated with improvements in ARDs (including other neurodegenerative diseases, vascular degeneration, osteoporosis, CKD, and T2D). Therefore, the upregulation of *A. muciniphila* abundance to prevent or combat ARDs in the older adult population through relatively safe treatments (e.g., diets that increase *A. muciniphila* abundance) may be a promising strategy. This initiative may help reduce the incidence of ARDs and control disease progression, thereby supporting healthy aging.

## Mechanisms of *A. muciniphila* against aging and age-related diseases

**Figure 1.**
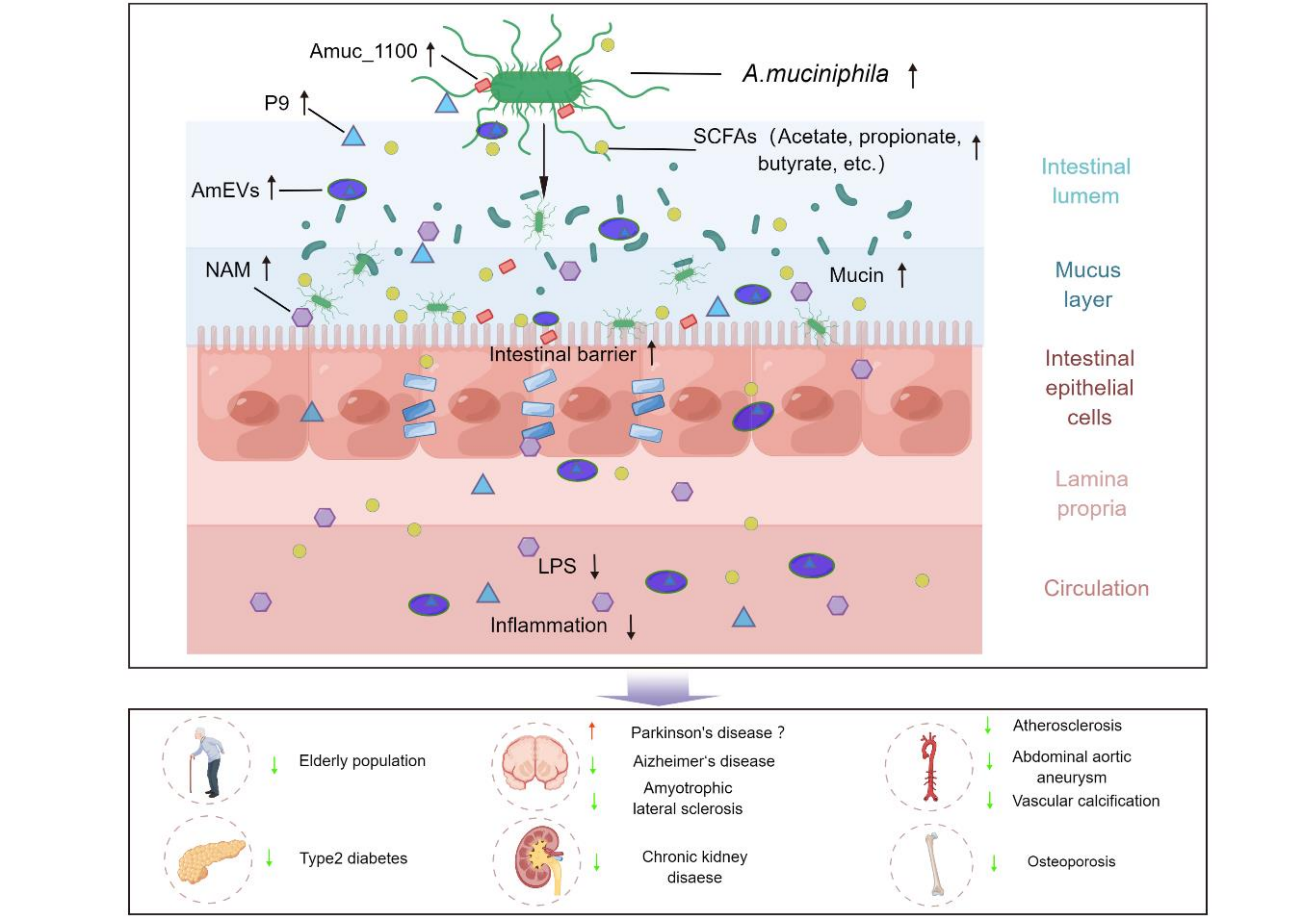
**Potential effects and major mechanisms of *A. muciniphila* on aging and ARDs**. By Figdraw (www.figdraw.com). Supplementation with *A. muciniphila* may be an effective project for anti-aging and alleviating ARDs, which is mainly through increasing the flow of metabolites such as SCFA and NAMs, restraining systemic inflammation, and secreting functional proteins and extracellular vesicles targeting to the extrenteral organs. However, the abundance of *A. muciniphila* is elevated in the gut of Parkinson's patients, but whether supplementation with *A. muciniphila* exacerbates the progression of Parkinson remains controversial. *A. muciniphila, Akkermansia muciniphila*; ARDs, Ageing-related diseases; SCFAs, Short-chain fatty acids; AmEVs, *A. muciniphila* -derived extracellular vesicles; NAM, Nicotinamide; LPS, Lipopolysaccharide.

Given the great potential of *A. muciniphila* in maintaining healthy aging, it is particularly important to explore its anti-aging mechanisms as this may lead to the development of more efficient and convenient anti-aging strategies. There are several main models based on the available evidence (Fig. 1).

### Increase metabolites in circulation

*A. muciniphila* ferments mucins or remodels the intestinal microbial community, thereby increasing the concentration of SCFAs in feces and serum[99-101]. However, it is worth noting that a reduction in SCFAs in the gut is one of the changes associated with aging [102]. The role of SCFAs in ARDs has been widely reported[103]. For example, the plasma concentrations of SCFAs were positively correlated with insulin sensitivity and fasting GLP-1 concentrations [104, 105], and supplementation with exogenous SCFAs, particularly butyrate, improved hyperglycemia and insulin resistance in T2D mice [106]. Acetic and propionic acids can regulate blood pressure regulators by stimulating Olfr78 to raise GPR41 levels or activate GPR41 to lower blood pressure [107]. Similarly, a range of evidence reveals the benefits of SCFAs for neurodegenerative diseases. The effects of sodium butyrate have been reported to include reduced motor dysfunction, oxidative stress, and neuroinflammation and increased striatal dopamine levels [108]. Notably, sodium butyrate protects dopaminergic neurons by upregulating genes involved in DNA repair and reversing damage caused by alpha-synuclein [109]. Furthermore, sodium butyrate and valproic acid ameliorate the behavioral deficits in AD mice by increasing histone acetylation and inhibiting glycogen synthase kinase-3β activity, respectively [110, 111]. In ALS mice, the number of butyrate-producing bacteria was reduced, whereas supplementation with 2% butyrate resulted in a delayed onset of ALS-related symptoms and increased lifespan [112]. A recent study showed that acetic acid replicates the ameliorative effects of *A. muciniphila* on ARDs, thereby extending the healthy lifespan of *Caenorhabditis elegans* and mice [28]. Based on the above evidence, it is reasonable to infer that the ability of *A. muciniphila* to produce SCFAs contributes to its beneficial effects in aging and ARDs.

Moreover, NAM production may be an integral part of the potential antiaging mechanism of *A. muciniphila*. A pivotal study found that NAM was reduced in patients with ALS and that exogenous supplementation of *A. muciniphila* and NAM in ALS mice increased the serum and cerebrospinal fluid levels of NAM, leading to remission of ALS [66]. Despite the lack of large-scale supporting data, this finding expands ideas for studying the therapeutic and pathological mechanisms of ALS.

### Maintenance of intestinal barrier

Gut physiology changes with age and preventing these changes can reduce microbiota dysbiosis and prolong lifespan [113, 114]. For example, the mucin production capacity of older-aged mice is drastically reduced and only a thin layer of mucus can be formed in the intestine [115]. When this layer is lost, harmful microorganisms can come into direct contact with the intestinal epithelium, disrupting the intestinal barrier and eventually causing systemic hypo-inflammation [116]. However, chronic inflammation is closely associated with aging and frailty and is a risk factor for ARDs [117]. Disruption of the intestinal barrier can also contribute in part to the progression of some ARDs, such as neurodegeneration, osteoporosis, and CKD [118-121]. Therefore, maintaining gut microbial homeostasis and intestinal permeability is necessary for healthy aging.

It is well known that most current studies support the benefits of *A. muciniphila* in reducing host endotoxemia and improving intestinal permeability. First, oral administration of live *A. muciniphila* may enhance the intestinal barrier by reinforcing the mucus and epithelial barriers and upregulating the expression of tight junction proteins in the intestine [50, 91]. In vitro experiments revealed that *A. muciniphila* can adhere to the surface of intestinal epithelial cells and directly reinforce the integrity of the intestinal epithelial cell monolayer [122]. Additionally, SCFAs of bacterial origin, such as butyric acid, have also been shown to protect the intestinal barrier [123, 124]. In addition, live *A. muciniphila*, as well as pasteurized *A. muciniphila*, Amuc_1100 on the outer membrane of *A. muciniphila*, and AmEVs were found to improve the intestinal barrier function [29, 125, 126].

Although overwhelming evidence supports the protective effect of *A. muciniphila* on the intestinal barrier, its applicability in all settings remains to be confirmed. This is because although one case report described no significant effect of *A. muciniphila* on the gastrointestinal tract, it had highly colonized in the intestine of 2 patients [127]. In contrast, *A. muciniphila* abundance was associated with more severe intestinal inflammation in a dextran sulfate sodium-induced colitis model and a mouse model of spontaneous colitis [72, 73]. Therefore, based on this uncertainty, administering more direct intestinal barrier protection measures, rather than simply supplementing with *A. muciniphila*, may be a safer and more effective anti-aging strategy.

### Secretion of functional proteins and AmEVs

To fully explain how the cell-free supernatant of *A. muciniphila* increased systemic GLP-1 secretion, Yoon et al. identified an 84 kDa functional protein named P9 [30]. P9 promoted metabolic health in obese mice when administered orally or intraperitoneally [128]. Mechanistically, the regulatory effect of P9 on glucose homeostasis is dependent on the presence of IL6, as it is ineffective in IL6 knockout mice [30]. However, whether P9 is a specific secretory protein of *A. muciniphila* and its effects on intestinal L cells are unclear. Nevertheless, this study revealed new ways in which *A. muciniphila* improves glucose tolerance and provided new insights into the mechanism of action of *A. muciniphila*.

In addition, AmEVs may be a potential mechanism by which *A. muciniphila* exerts its anti-ARD effects. In a mouse model, pretreatment with GW4869, a neutral sphingomyelinase (nSMase) inhibitor capable of impairing extracellular vesicle release, abolished the anti-osteoporotic effects of *A. muciniphila* [23]. Furthermore, AmEVs administered orally, rectally, or intravenously were delivered to the tibia and femur of mice within 1 h and exerted anti-osteoporotic effects *in vivo* [23]. The inhibitory effects of AmEVs on osteogenesis and osteoclastogenesis were further confirmed in vitro [23]. This evidence indicates the anti-osteoporotic effects of AmEVs. However, it remains unclear which components of AmEVs (miRNAs, proteins, etc.) play a key role. Therefore, subsequent studies are needed to follow this direction closely, which will help in understanding *A. muciniphila* more deeply and introduce new ideas for the management of osteoporosis.

## Conclusions and perspectives

Convincing evidence has supported a strong link between *A. muciniphila* and aging [129]. Although there may be differences in the effects of *A. muciniphila* on ARDs, it is clear that more evidence points to beneficial aspects (Table 1). However, rather than being anti-aging, *A. muciniphila* may maintain healthy aging. After all, *A. muciniphila* remains at a high level of abundance in healthy older adult populations (especially centenarians) [130, 131]. Thus, the abundance of *A. muciniphila* may only be reduced by the interaction between the organism and gut microbiota when the body becomes brittle, or ARDs appear during aging. However, whether unhealthy aging or ARDs can be avoided by early supplementation of *A. muciniphila* in humans to prevent a decline in its abundance is difficult to determine currently. However, several key concerns remain unresolved. First, the vast majority of ideas about *A. muciniphila* against ARDs have been proposed based on animal models. Given the unavoidable differences in genetics and the external environment between animal models and human diseases, the applicability of the benefits of *A. muciniphila* in humans is yet to be proven. Another reason the next generation of probiotics, including *A. muciniphila*, are not commercially available is that their safety evaluation does not meet the requirements of novel food regulations [132]. However, recent clinical studies have shown that *A. muciniphila* can be safely administered to patients with T2D with some efficacy [133]. This discovery paves the way for further clinical studies on *A. muciniphila*. Third, the potential impact of *A. muciniphila* on ARDs is not entirely consistent. For example, *A. muciniphila* colonization in the gut of PD patients is increased and correlates with disease progression [70]. Finally, the abundance of *A. muciniphila* in its natural state varies among human populations, making it tolerated differently by different groups. Therefore, the amount of *A. muciniphila* supplementation should also be tailored to different populations, especially considering the potential adverse effects of excessive amounts of *A. muciniphila* [134].

**Table 1:** Association of *Akkermansia muciniphila* with age-related diseases.

Age-related disease	Change in *Akkermansia muciniphila* abundance in the disease group compared to the healthy control group	Efficacy after intervention with *Akkermansia muciniphila*	Potential mechanisms	Ref.
**Atherosclerosis**	Downward (mice)	Beneficial for disease (mice)	Maintain the intestinal barrier	[50]
**Abdominal aortic aneurysm**	Downward (mice)	Beneficial for disease (mice)	Restoration of intestinal microbiota diversity and modulation of peripheral immune factor expression	[54, 55]
**Vascular calcification**	NA	Beneficial for disease (mice)	Improving the imbalance of the intestinal microbiota and maintaining the intestinal barrier	[58]
**Alzheimer's disease**	NA	Beneficial for disease (rat and mice)	Regulation of the composition of the intestinal microbiota	[64, 65]
**Amyotrophic lateral sclerosis**	Downward (mice)	Beneficial for disease (mice)	Elevated systemic and cerebrospinal fluid levels of nicotinamide	[66]
**Parkinson's disease**	Upward (human)	Does not cause motor deficiency	NA	[67, 68, 70]
**Osteoporosis**	Downward (human, rat and mice)	Beneficial for disease (mice)	Secretion of extracellular vesicles	[23, 78-80]
**Chronic kidney disease**	Downward (human)	Beneficial for disease (rat)	Improving the imbalance of the intestinal microbiota and maintaining the intestinal barrier	[86, 87]
**Type 2 diabetes**	Downward (human and mice)	Beneficial for disease (human and mice)	Maintaining the intestinal barrier and secretion of a glucagon-like peptide-1-inducing protein	[29, 30, 91-94, 126]

Although the utility of *A. muciniphila* still has a long way to go before it crosses over from preclinical studies to clinical applications, its future in maintaining healthy aging appears to be bright. In this context, we believe that future studies on *A. muciniphila* should focus on these aspects. First, a comprehensive understanding of the differences in the effects and detailed mechanisms of action of *A. muciniphila* and its derived active components is required. In particular, the mechanisms specific to antiaging agents or ARDs need to be elucidated. Furthermore, more attention should be paid to the interaction between *A. muciniphila* and symbiotic bacteria, as this is promising for explaining the differences in the effects of *A. muciniphila* on human diseases.
